# How COVID-19 impacted surplus food redistribution in the Netherlands: An explorative study

**DOI:** 10.1007/s12571-022-01291-y

**Published:** 2022-05-21

**Authors:** Madhura Rao, Aalt Bast, Alie de Boer

**Affiliations:** 1 Food Claims Centre Venlo, Maastricht University, Campus Venlo, Maastricht, the Netherlands; 2 University College Venlo, Maastricht University, Campus Venlo, Maastricht, the Netherlands; 3 Department of Pharmacology & Toxicology, Faculty of Health, Medicine and Life Sciences, Maastricht University, Maastricht, the Netherlands

**Keywords:** Food aid, Surplus food, Food waste, Food security, Qualitative research

## Abstract

The COVID-19 pandemic has been detrimental to food security globally. The Netherlands, despite its advanced stage of development, saw a surge in food insecurity among its most vulnerable citizens. Dutch food aid is managed by private charities and social organisations that often aim to address the problems of food insecurity and food waste by redistributing surplus food that is safe to consume. This paper investigates how the pandemic impacted surplus food redistribution in the country by employing an Exploratory-Descriptive-Qualitative approach. This is done by analysing data from interviews with relevant stakeholders involved in redistributing surplus food in the Netherlands as well as media reports on the topic. Our findings indicate that the interviewed organisations experienced drastic fluctuations in supply and demand. To cope with these changes, rapid organisational and supply chain innovation was observed. Next to this, there seems to have been disproportionate negative impact on smaller charities in comparison to bigger, better established organisations. Based on our findings, we discuss what the future of surplus food distribution in the Netherlands might look like and why changes made during the pandemic must be well documented and carefully analysed.

## Introduction

At the time of writing this paper, more than two years have passed since the SARS-CoV-2 virus made its presence felt around the world. Based on reports from the media, national governments, and international organisations, it is widely known that the crisis has been detrimental for food security globally (Devereux et al., 2020; Laborde et al., 2020; Swinnen & McDermott, 2020). The Netherlands, despite its advanced stage of development, is home to close to a million people living below the poverty threshold (Centraal Bureau voor de Statistiek, 2021). This threshold is set at 60% of the national median disposable household income allocated to all persons belonging to one household (Centraal Bureau voor de Statistiek, 2021).^1^ Poverty in the Netherlands is viewed and calculated in relation to the general level of prosperity of the society. The State ensures that all citizens have access to housing, health care, and food in quantities sufficient to prevent hunger. However, belonging to a low income household considerably affects individuals’ ability to participate in society and live a fulfilling life (Centraal Bureau voor de Statistiek, 2021). In the context of food security, this could mean that individuals belonging to low-income households are unable to access nutritious or high-quality foods in sufficient quantities. Refugees, single-parent families, citizens receiving social assistance, individuals with lower levels of education, and those in the 55–65 age group were found to be the most vulnerable to being affected by poverty (Centraal Bureau voor de Statistiek, 2021).

For several individuals belonging to low income households in the Netherlands, food aid or discounted food packages from various privately run organisations across the country were a vital source of food security before the onset of the pandemic (van der Horst et al., 2020). The economic hurdles brought about by the crisis are likely to have made these resources even more crucial. The objective of this paper is to examine how the work of these organisations was impacted by the pandemic.

Surplus food is an important resource for organisations that assist food insecure individuals in the Netherlands. By redistributing this food while it is still safe to consume, these organisations seek to address the highly pertinent issues of food insecurity and food waste. Surplus food is defined as ‘edible food that is produced, manufactured, retailed or served but for various reasons is not sold to or consumed by the intended customer’ (Garrone et al., 2014a). Redistribution in this context is to be understood as the distribution (of food) in a way that is different from what was originally planned. Although surplus redistribution alone cannot sufficiently improve food security and reduce food loss and waste, it remains an important way to prevent food from entering waste streams (Midgley, 2014). Ideally, from a resource efficiency perspective, the production of excess food should be prevented whenever this is possible (Papargyropoulou et al., 2014). When prevention is not possible due to technical, economic, regulatory, or other reasons, redistributing this surplus among consumers should be the next priority (Baglioni et al., 2017; Garrone et al., 2016). This can take on several different forms such as reusing surplus food in primary markets by repackaging or remanufacturing, selling it in secondary markets at discounted prices, or redistributing it to food insecure people (Baglioni et al., 2017). This paper looks at selling surplus food in secondary markets at discounted prices and redistributing it to food insecure people in the context of food redistribution operations in the Netherlands.

It is estimated that between 1.77 and 2.55 million tonnes of food is wasted in the Netherlands every year (Soethoudt & Burgh, 2017). Between 2009 and 2015, the Dutch government aimed to reduce the amount of food waste by 20% (Soethoudt & Burgh, 2017). Despite creating several initiatives, businesses and policy makers have not been able to achieve the desired reduction (Soethoudt & Burgh, 2017). At the same time, close to 1 million people out of the 17 million population belong to low-income households and require assistance in gaining access to sufficient quantities of nutritious food (Centraal Bureau voor de Statistiek, 2021; van der Horst et al., 2020). Therefore, redistributing food that is safe to consume and nutritious is an important way to reduce food loss while improving food security in the Netherlands. Globally, the issues of food waste and food insecurity have been exacerbated by the COVID-19 pandemic (Galanakis, 2020). Although national figures indicating the impact of the pandemic on food waste and food insecurity levels are not available at the time of writing this paper, we can speculate that the social and economic disruptions that the country experienced during this time negatively affected both issues.

The first officially recognised COVID-19 infection in the country occurred on February 27, 2020. On March 15, the first ‘intelligent’ lockdown was announced wherein the government sought to focus on moral appeal and self-discipline as opposed to repression to implement lockdown measures (Kuiper et al., 2020). The months that followed saw the lockdown getting extended, often with stricter measures.^2^ Public gatherings, restaurants, and much of social life were heavily restricted in the country in the years 2020 and 2021. These restrictions negatively impacted the economy and in turn, individuals whose livelihood depended on affected sectors. The crisis magnified the vulnerability of the most deprived in Dutch society, who saw their access to healthy and nutritious food reduced (Candel & de Zwarte, 2020). A disruption in the operations of charity organisations is likely to impact those who are dependent on them to meet their nutritional needs as well as businesses who relied on them to manage their surpluses.

In recent years, improving food security in high income countries via surplus redistribution and donation has received much interest from a variety of academic fields. Existing literature examines this phenomena in Dutch (Neter et al., 2016, 2018; Rao et al., 2021; van der Horst et al., 2014, 2020; van der Velde et al., 2019), European (Alexander & Smaje, 2008; De Boeck et al., 2017; De Pieri et al., 2017; Garrone et al., 2014b; Lambie-Mumford, 2016, 2017), and global contexts (Caraher & Furey, 2017; Lambie-Mumford & Dowler, 2015; Riches, 2018; Silvasti & Riches, 2014). By providing the first empirical evidence on how food charity and surplus redistribution in the Netherlands was affected by COVID-19, this paper contributes towards the systematic documentation of the pandemic’s impact on the food system and furthers the discourse on the role of private organisations in improving food security and preventing food waste.

## Methods

Given that the pandemic is ongoing at the time of writing this paper and no previous empirical studies examining the impact of COVID-19 disruptions on food surplus redistribution in the Netherlands have been published, the Exploratory-Descriptive Qualitative (EDQ) method described by Hunter and Howes (2020) was seen as the most suitable method to conduct this research. The EDQ method allowed us to use a purposeful sampling strategy, collect data through semi-structured interviews, and generate themes from the analysed data. It offered a broad-ranging and systematic approach to understanding and describing a previously unexplored area of social life.

This research is part of a broader study examining food waste valorisation in the Netherlands (manuscript in preparation) and received an ethics approval from the Ethics Review Committee Inner City faculties of Maastricht University. All participants interviewed for the larger study were asked about the impact of the pandemic on their operations. Eight participants involved with five organisations redistributing surplus food in the Netherlands provided detailed insights on the issue and were therefore included in this study. Participants were asked how the pandemic and the measures implemented to contain it affected their operations. They were then requested to elaborate on whether they expected these changes to have any long and short-term consequences for their work. Furthermore, they were asked to reflect on the impacts on food security and food waste. Based on the provided answers, we asked additional questions to allow participants to further elaborate on their experiences with food surplus redistribution during this period. Most participants were in senior executive positions in their respective organisations and were directly involved in making pandemic-related changes to their operations. As a result, they were able to share vivid accounts on the subject. Additionally, the five included organisations are diverse in terms of size, mode of operation, and financing. Some have several branches throughout the country while others operate as single organisations. While two offer food parcels containing groceries, others serve prepared meals. Sources of financial support include the national government, local municipal corporations, and private donors. Therefore, despite the limited sample size, our dataset is comprehensive and offers ample insight into the subject. Table 1 provides information about each participant’s background.

**Table 1:** Study participants

**Participant**	**Position**	**Organisation**
P1	Board member	A well-established network of food charities in the Netherlands with 150 + branches and 10 distribution centres across the country. This organisation donates surplus food to food insecure individuals by composing food parcels containing a variety of food products and ingredients. This organisation will be referred to as ‘Charity Network’ in this paper
P2	Board member	
P3	Volunteer recruited for additional procurement during the pandemic	
P4	Chief operating officer	A non-profit organisation providing logistics support to businesses that wish to donate surplus food to soup kitchens and social restaurants. This organisation will be referred to as ‘Logistics Help’ in this paper
P5	Project leader and volunteer	A non-profit that prepares free meals from salvaged food from supermarkets and catering at eleven locations in the country. This organisation will be referred to as ‘Free Meals’ in this paper
P6	Key account manager	A digital platform that offers customers surplus food from retail stores and catering at discounted prices. This organisation will be referred to as ‘Pay It Forward’ in this paper
P7	Co-founder and project manager	A non-profit organisation linking companies with food surpluses to social organizations that prepare meals for vulnerable local residents. This organisation will be referred to as ‘Surplus Hero’ in this paper
P8	Founding member and volunteer	An unregulated charity shop with a refrigerator placed outside its premises for anyone to drop off or pick-up surplus food. This organisation will be referred to as ‘Community Fridge’ in this paper

For data collection, we used a purposive, snowballing sampling strategy. Participants were contacted via email by the first author. In line with the lockdown measures, interviews were conducted online via Zoom between August and November 2020 by the first author. During this period, six to nine months had passed since the virus was first detected in the country. The lockdown measures which were introduced in March and then reinforced more strictly in following months had been relaxed. August to November 2020 also saw a steep increase in the number of infected people. Interview questions were framed keeping these developments in mind. It is likely that these trends also informed how participants perceived the pandemic and its impact on their work. The interviews were in-depth, semi-structured and were conducted in the English language on a one-on-one basis. With the participants’ consent, interviews were recorded and were later transcribed *verbatim* using the Otter.ai software. Transcripts were sent to participants via email to allow them to do a factual check. After all participants consented to the transcripts being used for this research, they were analysed using the Atlas.ti software (version 8.4).

In line with the explorative nature of this research, the data were analysed through an inductive approach. The first and third author undertook data analysis independently between December 2020 and February 2021 and later compared and consolidated their findings. The thematic analysis approach by Braun and Clarke (2006) was chosen because it offered theoretical freedom and high flexibility in the analysis of the collected data and fits well with the EDQ method (Hunter & Howes, 2020). The transcripts were first read on their own, allowing us to understand the premises of discussions and note the similarities and differences that arose. Then we used the in vivo coding method from Saldana (2021) to generate a first set of codes. This was followed by a second cycle of coding wherein codes that pertained to the same theme were grouped together. Finally, three themes were constructed from the codes. These themes are described in the next section. Wherever necessary, excerpts, quoted *verbatim*, unless modified to improve readability or ensure anonymity, have been used to underpin the findings.

To corroborate observations made from analysing data from the interviews, we used data triangulation as a validation strategy. Specifically, we employed the between-method triangulation strategy described by Flick (2004) because it allows us to use a secondary non-reactive data collection procedure to complement our primary reactive procedure of in-depth, semi-structured interviews. We did this by analysing Dutch media reports published between March and December 2020 about surplus food redistribution. Reports were obtained by searching the LexisNexis database (available via lexisnexis.nl) using ‘*voedseloverschotten’, ‘voedseldonatie’, ‘voedsel liefdadigheid’,* and *‘voedselhulp’* as keywords. Next to these keywords, names of the participants’ organisations were also searched on the database. Analysing the contents of these reports allowed us to gain greater rigor in our analysis and rule out rival hypotheses that could be constructed from the primary data. It also helped us in substantiating factual information such as timelines, quantities of surplus food available, and the strictness of lockdown measures which were mentioned by the study participants.

## Findings

### Changes in demand and supply

Most participants reported experiencing fluctuations in demand and supply for surplus food. P1 and P2 from the Charity Network were of the opinion that a rise in unemployment might mean that more people will approach their organisation for food parcels. P1 spoke about expectations regarding this in the near future: *“That (increase in demand) has just started. In the big cities, we have already seen an increase of 20—25%. In the countryside, it's hardly increased. But if you look at the news, there is an expectation of an increase in the number of unemployed people. We do expect a 50% increase in total.”*

The Central Agency for Statistics (Centraal Bureau voor de Statistiek) confirms that the pandemic has resulted in an unprecedented drop in the number of jobs and that the number of hours worked in the second quarter of 2020 was 6.1% lower than in the first quarter (Centraal Bureau voor de Statistiek, 2020). Observations by the FAO also indicate that owing to an increase in the number of people affected due to the surge in unemployment, food banks across the developed world are expecting a sharp rise in the demand for their services (FAO, 2020). Given this, it is not surprising that the Charity Network recruited additional volunteers to procure food from new sources. P3 was one such volunteer whose service to the organisation included identifying new sources of surplus food and financial support. In the initial months of the lockdown, P3 recalled experiencing an increase in supply from restaurants and caterers and a dip in the quantities received from supermarkets. It can be speculated that having to deal with the disruptions in their own supply chains made supermarkets reluctant to donate. However, at the time of the interview (six months since the start of the lockdown), P3 mentioned that most supermarkets were back on track and regular supply had resumed.

The Charity Network’s experience of an increase in demand was not shared by other small-scale organisations such as Free Meals and Surplus Hero. These organisations cook meals using surplus food and offer them to clients who eat together free of cost. Unlike the Charity Network, these organisations do not offer the option to pick up food parcels. Much like restaurants and catering organisations, both were compelled to stop receiving clients when lockdown measures were announced. P4 from Logistics Help informed us that their organisation works mostly with charities that cook meals and operations had to be halted until clients could be hosted for meals again. Despite a sharp fall in demand, Logistics Help received several offers from businesses that wished to donate food in the weeks that followed the lockdown.

Pay It Forward experienced a minor decrease in supply from supermarkets. P6 mentioned that fewer clients seemed to be willing to visit supermarkets to pick up the surplus parcels. Since supply and demand dropped in proportion to one another, the net result was that most of the surplus parcels offered though Pay It Forward were purchased. It is, however, important to note that not all participants or their organisations were able to keep an account of the demand and supply fluctuations. This is especially true for smaller organisations that depend on volunteers and therefore did not have the resources required to systematically analyse fluctuations. For instance, P5 from Free Meals mentioned: *“It is hard to tell (whether there was a change in demand) because communication took a different route. People normally pass by our community houses but now it is difficult to contact our guests. Some people totally isolated themselves. So, they might be in need of (food) donation but not feeling safe enough to actually open the door or pick it up. So yeah, it's hard to tell. We don't know the specific numbers and whether we actually surpassed normal amount (of meals provided) or not or if there was a higher demand than we could supply.”*

This fall in demand experienced by organisations that offer cooked meals is likely to be a reaction to the lockdown measures. Based on our findings and news reports that were analysed, it is unclear whether the supply of surplus food has increased in proportion to the demand for it.

### Alternative modes of operating

Most participants mentioned that their organisations developed alternative ways of operating in order to continue redistributing surplus food while following the lockdown measures. Some branches of the Charity Network had to halt their operations and offered clients EUR 30 vouchers per week in lieu of the weekly food parcels. P4 from Logistics Help noticed that some charities they worked with started delivering cooked meals to clients’ homes. Free Meals altered their operations in a similar manner and collaborated with professional cooks. P5 described the new way of operating as follows: *“We collaborated with restaurant kitchens that were closed and chefs who were at the moment unemployed and wanted to help us with cooking. They managed the operations in a very structured way; with as few people as possible while following the HACCP guidelines. They even used their own kitchens. From there on, we could just distribute meals to our clients. We were able to find their contact details and deliver meals instead of them stopping by for dinner”.* This collaboration exhibits innovation in not only the mode of delivery but also recruiting new volunteers, procuring a new working space, and establishing contact with former clients.

During this period, several community-led initiatives focused on food waste and food insecurity were started in the Netherlands. Some examples include Benefrietjes^3^, Aardappelberg^4^, Etenover.nl^5^, Kies Lokaal by Slowfood NL^6^, and Support Your Locals^7^. Pay It Forward and Surplus Hero collaborated with some of these initiatives through volunteering, advertising, and offering advice. Many initiatives that were started during this period were founded on an ad hoc basis and are not operational anymore. The lockdown measures left several farmers who normally supplied to hospitality and catering businesses with surplus fresh produce. Using its digital platform, Pay It Forward attempted to connect interested clients with this surplus. Describing the idea, P6 explained: *“What we did see is that suppliers to food service, mushroom farmers for instance, were left with excess produce. It was a huge pile that they suddenly got stuck with. There was real interest from people because they were sitting at home anyway. They were willing to go to a farmer and buy some mushrooms.”* In addition to having the time to carry out this exercise, other factors could have motivated consumers to buy directly from farmers. Consumers could have had an interest in helping farmers, supporting the local economy, purchasing food in a new way, and preventing food from going to waste.

Some participants and media reports indicated that social media platforms played an important role in helping surplus food redistribution continue during this time. For instance, before the pandemic, Community Fridge relied on donors taking notice and stocking the refrigerator on their own accord. This turned out to be an ineffective strategy when lockdown measures were implemented. Under the given circumstances, Community Fridge found Facebook to be a useful tool to ensure that they had a steady supply of food. P8 described their use of Facebook to remind donors (private individuals residing in the neighbourhood) to continue donating: *“I posted a message on Facebook saying please don’t forget about the refrigerator, especially now because we need to help each other. Then I saw that it was shared quite widely. And I think after that, the community became more actively involved in keeping the fridge full.”*

At the time of their interviews, participants were unable to comment on whether some changes made during this time could be incorporated into their organisations’ operations after the pandemic was over and normalcy had returned.

### Disproportionate impact on small organisations

When studying or reporting about surplus food redistribution in the Netherlands, the Charity Network receives much attention from scholars and the media. However, it is not the only organisation in the country working towards improving food security and preventing food waste. Smaller organisations, some of which have been included in this study, play a crucial role in reaching individuals who cannot avail help from the Charity network. However, due to their small size, their work seems to go unnoticed at the national level. This lack of attention and support had severe consequences for smaller charity organisations and their clients during the pandemic.

While the Charity Network, with its large number of volunteers, strong public and donor relations, and structured way of operating was able to benefit from governmental and private support, smaller charity organisation and their work were hardly mentioned in the media. As per the information provided by relevant study participants as well as studied reports, the Charity Network was helped by the Dutch government via monetary support that amounts to several million euros. As of December 2020, the organisation had not spent any part of this amount because it was able to sustain itself without additional help from the government.

In order to avail food parcels from the Charity Network, prospective clients are required to pass a number of criteria related to their income, expenditure, status as a resident, among others. Smaller organisations tend to be less stringent about these criteria and as a result provide aid to those who do not qualify for receiving donations from the Charity Network or those who require additional help next to the parcels received from the Charity Network. P5 from Free Meals mentioned: *“We had to close our locations in March and that was a pain to our hearts. It really felt wrong to do so. We provide critical help for many people, even those who are undocumented immigrants.”*

Another major difference between the Charity Network and smaller organisations is that branches of the Charity Network often operate out of premises that are either owned or rented by the parent organisation. As informed by various study participants, smaller non-profits operate out of community centres either by paying a nominal fee or free of cost. When community centres were shut down in line with the lockdown measures, these organisations were unable to continue with their work. A lack of operating premises meant that these organisations could not provide support to clients even if there was surplus food available. P5 stated this as a reason for halting operations at Free Meals: *“The community houses told us that we could no longer run the operations on their premises. They were instructed by the municipality to not facilitate any group meetings.”*

Multiple study participants speculated that had a certain portion of government funds or support from retail or other donors been directed towards smaller organisations, they could have continued to provide critical help to at least a part of the several food insecure individuals who depend on them. P7 from Surplus Hero expressed disappointment with regard to the Charity Network receiving more attention compared to other organisations: *“We saw that a lot of supermarkets are donating to the Charity Network. They only reach 10% of the poor people but everybody is focused only on them. Other social organisations are open for everybody. The 90% who cannot go to the Charity Network, they are helped by us. With the way we work, they not only have a free meal but also social contact. The Charity Network, of course, is doing good work but they only reach a small percentage of the people in need.”*

Based on personal exchanges with supermarkets to urge them to resume donations after the lockdowns were eased, P7 wondered whether supermarkets were using the pandemic as an excuse to not donate: *“In the beginning all the supermarkets were really stressed and there was not much left. However, after a while they had surplus but still, they mentioned Corona virus or Corona crisis as a reason to just keep the door shut. A lot of the donation relations stopped for us.”*

Overall, our findings indicate that smaller non-profit organisations redistributing surplus food have been severely impacted by the pandemic. In comparison, larger, better established organisations such as the Charity Network, Red Cross, and the Salvation Army were able to continue providing food aid either by purchasing food products or redistributing surplus (Het Leger des Heils, n.d.; Rode Kruis Nederland, 2020). We did not find any media reports discussing this issue, but we did observe that the Charity Network and other large organisations received a lot more media coverage compared to their smaller counterparts.

## Discussion

Although explorative in nature, our findings indicate that the COVID-19 pandemic has brought about several changes in the way surplus food is redistributed in the Netherlands. As per the findings of this study, increase in demand for food aid, new modes of operating for redistribution organisations, and disproportionate negative impact on smaller charities are the most evident consequences of the pandemic on surplus food redistribution. The shockwaves created as a result of the drastic changes in food supply chains and increased demand for food aid have pushed surplus redistribution operations to transform themselves at an extraordinary pace. Several of these changes and new practices could have the potential to help the food redistribution system build back better.

Some changes presented in Sect. 3 of this paper have the potential to have a positive impact in the long time if adopted systematically even after the pandemic is over. For instance, NGOs and social enterprises could help connect consumers with surplus from farmers or wholesale suppliers. The timely consumption of this food could reduce its likelihood of ending up as waste. Charities and soup kitchens collaborating with professional cooks could help improve the safety and quality of meals provided. If redistribution organisations continue to deliver food parcels or meals even after the pandemic is over, food aid could become more accessible to individuals living with disabilities. Smaller charities could gain social and financial support by strengthening their social media presence. To benefit from the changes that have taken place during the pandemic, it is vital that they are well documented. Larger organisations with sufficient resources can help with this process.

Conversely, changes made during the pandemic must also be critically examined to assess whether they are indeed sustainable in the long term. For instance, disrupted logistics and lesser focus on food waste could have made surplus food unavailable during this period. The Charity Network’s plan to set up a foundation that purchases food instead of procuring surplus could be a result of this. On the one hand, surplus food which is available in abundance in the country might end up as waste if charities start purchasing food via conventional supply chains. Instead of allocating resources for purchasing food, they could be used for improving the process and logistics of redistributing surplus food. On the other hand, critical scholars have suggested that using surplus to feed food insecure citizens is a demeaning practice. Caraher and Furey (2017) suggest that this practice takes away citizens’ ability to choose food in socially acceptable and dignified ways. It allows others to make the choice on their behalf by repurposing food that is not considered saleable by the retail sector (Caraher & Furey, 2017). A way to find middle ground could be platforms such as Pay It Forward partnering with the government to provide surplus from retail and service to food insecure individuals free of cost. The fact that this surplus is available to and utilized by food secure individuals as well, albeit at a price, might take away the stigma attached to consuming surplus food.

Another significant finding of this paper has been that smaller organisations engaged in surplus redistribution have been largely excluded from public and private support. While it is understandable that well-established organisations are seen as more reliable due to their track record and visibility in the media, they cannot improve food security and reduce food waste alone. As shown in Sect. 3.3, smaller organisations and community-led initiatives have the potential to reach sections of the society that are inaccessible to large organisations such as the Charity Network. Based on responses of our study participants, it can be speculated that factors such as being an undocumented migrant, cultural preferences, previous experiences with food aid, and the requirement to disclose personal information might prevent some individuals from approaching larger organisations. Support in the form of monetary help or donating surplus food can provide a much-needed impetus to smaller organisations.

Our arguments supporting equitable redistribution of surplus food brings to the forefront criticism regarding private entities taking on the role of providing aid to food insecure citizens. The existence of such charities in developed countries has attracted the critique of several researchers who see it as a failure of the State welfare mechanisms (Dowler & O’Connor, 2012; Lambie-Mumford, 2017; Poppendieck, 1999; Riches & Silvasti, 2014). As put forth by van der Horst et al. (2020), hunger brings with it a moral imperative to act. Discussions around whose responsibility it is to provide aid are not an immediate concern (van der Horst et al., 2020). However, the Netherlands’ dependence on private organisations to provide aid to the food insecure and alleviate the problem of food waste is not a reaction to the pandemic. Over the course of the last two decades, private charities have become an important part of looking for a solution to both problems in the Netherlands. When private entities are in charge of addressing such systematic and widespread issues, gaps may emerge in the State’s welfare provision and structural problems may go unnoticed or be neglected (van der Horst et al., 2020).

As per Candel and de Zwarte (2020), the COVID-19 crisis shows that the privatisation of food aid harms the most vulnerable in society. They implore the State to view the outcomes of the crisis as an opportunity to make structural improvements to reduce inequalities and food insecurity. Codifying the right-to-food as a formal law would be a step in this direction. Our work affords relevant actors in the Netherlands as well as other high-income countries with similar food redistribution systems an opportunity to address the systemic inefficiencies in food security and food waste management that were brought to the forefront by the pandemic.

## Limitations and future directions

While the findings discussed in this paper are intriguing, they represent only a first foray into understanding how the COVID-19 pandemic might have impacted surplus food redistribution. Being exploratory in its approach, our study presents some limitations; the first one being the limited sample size. Using this paper as a foundation, an in-depth study with a larger sample size can help validate our findings. Collecting and analysing data on how doners of the surplus food, for example retail, catering, and wholesale establishments, experienced the pandemic could provide a more complete and multi-perspective picture of surplus food redistribution during this period.

A second limitation is that we were unable to cite or provide information about the media reports analysed for data triangulation. This was done so as to maintain anonymity and protect study participants. The need to maintain anonymity posed restrictions on the literature that could be cited in this paper as well. We exclude several papers focusing on specific organisations included in this study to ensure that our participants are protected.

However, no previous studies have presented empirical findings on the topic, and this makes our study an important first step in identifying how COVID-19 affected surplus food redistribution in the country. Once the pandemic subsides, it could be interesting to explore whether any changes made during this period have been incorporated into the regular operations of relevant organisations. This study could also be replicated in other regions of the world to infer how surplus redistribution was impacted on a global scale.

## Concluding remarks

The aim of this paper was to identify how the COVID-19 pandemic impacted surplus redistribution in the Netherlands. Based on our findings, we conclude that the crisis has led to an increased pressure on food charities that provide aid to food insecure individuals by redistributing surplus food. At the moment of writing this paper, it is unclear whether the supply of surplus food has increased in proportion to the demand. Next to this, smaller organisations in the country received little to no support from the government and the retail sector, both of whom focused on larger, better- established charities. Lastly, the pandemic has led to new ways to redistribute surplus food. Some of these new practices could potentially help in addressing the issues of food waste and food insecurity in a post-pandemic world as well.

Governments, legislative bodies, and civil societies should view the consequences of the pandemic as an opportunity to initiate a conversation regarding the private governance of surplus food redistribution. Given that food waste and food insecurity are significant global problems, stakeholders should start thinking of ways to use this surplus to help feed those who are in need without compromising their dignity.

## Data Availability

The corresponding author can be contacted to request a copy of the code book.

## Footnotes

1 In 2020, EUR 1020 was considered to be the minimum disposable income required by a single person household for subsistence (Centraal Bureau voor de Statistiek, 2021)

2 A chronological account of the COVID-19 measures in the Netherlands from February 2020 to February 2021 can be found here: https://perma.cc/5YTE-37UV

3 Benefrietjes was an initiative to help use up the surplus stock of potatoes in the country by encouraging consumers to buy more fries

4 Aardappelberg, translating to ‘potato mountain’, was an initiative wherein consumers could purchase surplus potatoes from farmers in various Dutch cities at a reduced price.

5 Etenover.nl was a digital platform connecting consumers with surplus from catering and hospitality suppliers.

6 Kies Lokaal by Slowfood NL is an interactive map collating multiple food security and food waste prevention initiatives throughout the Netherlands.

7 Support your locals is a website that helps small food businesses by allowing visitors to 542 search for local entrepreneurs in their neighbourhoods and purchase from them directly.
